# Metabolomics analysis reveals enhanced salt tolerance in maize through exogenous Valine-Threonine-Isoleucine-Aspartic acid application

**DOI:** 10.3389/fpls.2024.1374142

**Published:** 2024-05-17

**Authors:** Kaihua Wu, Xiaoyan Liang, Xiu Zhang, Guoping Yang, Huaxiao Wang, Yining Xia, Shumila Ishfaq, Hongfei Ji, Yuxi Qi, Wei Guo

**Affiliations:** ^1^North Minzu University, Ningxia Key Laboratory for the Development and Application of Microbial Resources in Extreme Environments, Yinchuan, China; ^2^Institute of Food Science and Technology, Chinese Academy of Agricultural Sciences/Key Laboratory of Agro-products Quality and Safety Control in Storage and Transport Process, Ministry of Agriculture and Rural Affairs, Beijing, China; ^3^Gembloux Agro-Bio Tech, Liege University, Laboratory of Integrated and Urban Plant Pathology, Gembloux, Belgium

**Keywords:** exogenous, maize, metabolomics, salt stress, short peptide

## Abstract

Salt stress is a well-known abiotic constraint that hampers crop productivity, affecting more than 424 million hectares of topsoil worldwide. Applying plant growth regulators externally has proven effective in enhancing crop resilience to salt stress. Previous metabolomics studies revealed an accumulation of Valine-Threonine-Isoleucine-Aspartic acid (VTID) in salt-stressed maize seedlings, suggesting its potential to assist maize adaptation to salt stress. To explore the effectiveness of VTID in enhancing salt tolerance in maize, 10 nM VTID was applied to salt-stressed maize seedlings. The results showed a remarkable 152.29% increase in plant height and a 122.40% increase in fresh weight compared to salt-stressed seedlings. Moreover, the addition of VTID enhanced the activity of antioxidant enzymes, specifically superoxide dismutase (SOD) and catalase (CAT), while reducing the level of malondialdehyde (MDA), a marker of oxidative stress. Additionally, VTID supplementation resulted in a significant increase in osmoregulatory substances such as proline. Metabolomic analysis revealed substantial changes in the metabolite profile of maize seedlings when treated with VTID during salt stress. Differential metabolites (DMs) analysis revealed that the identified DMs primarily belonged to lipids and lipid-like molecules. The receiver operating characteristic curve and linear regression analysis determined a correlation between isodolichantoside and the height of maize seedlings under salt-stress conditions. In conclusion, these findings validate that VTID effectively regulates tolerance in maize seedlings and offers valuable insights into the potential of short peptides for mitigating salt stress.

## 1 Introduction

Salt stress is considered one of the most severe abiotic challenges, exerting widespread impacts on plant growth, development, and productivity worldwide (Xiao and Zhou, 2023). According to the Global Map of Salt-Affected Soils (GSASmap), a vast area of both topsoil (0-30 cm) and subsoil (30-100 cm) has been identified as salt-affected, encompassing 424 million hectares and 833 million hectares, respectively (Food and Agriculture Organization, 2023). Saline soils contain high levels of soluble salts, and pose significant threats to plants by inducing toxicity and disrupting nutrient balance, biodiversity, and water availability in the soil (Hu et al., 2022). Excessive concentrations of cations and anions (Na^+^ and Cl^-^) in the soil trigger physiological and biochemical changes in plants, resulting in reduced cellular water content and increased generation of reactive oxygen species (ROS) (Qin et al., 2022). In response to salt stress, plants employ various non-enzymatic and enzymatic mechanisms. During stress, plants accumulate osmoregulatory substances, such as proline, soluble sugars, soluble proteins, and betaine, which help to maintain physiological functions by reducing cellular osmotic potential (Ru et al., 2023). Additionally, the activities of well-known antioxidases, such as superoxide dismutase (SOD), peroxidase (POD), and catalase (CAT) are upregulated to counteract ROS induced by salt stress (Xia et al., 2014).

Besides, several exogenous plant growth regulators (PGRs), including melatonin, polyamines, brassinosteroids, and steroid hormones, have been documented to enhance stress resistance by modulating enzyme activity and metabolic levels (Gai et al., 2020). The application of 100 µM melatonin has been found to reduce salt stress by promoting root, shoot length, fresh and dry weight, increasing chlorophyll contents, and inhibiting excessive production of oxidative stress markers (Khan et al., 2024). As a type of polyamine, 0.3 mM spermidine was found to recover the Zoysiagrass seedlings’ growth by increasing antioxidant enzyme activities and decreasing H_2_O_2_ and malondialdehyde (MDA) levels (Li et al., 2016). Exogenous application of 10^−9^ M 24-epibrassinolide on rice variety Pusa Basmati 1 showed an improvement in plant growth, levels of protein and proline content, antioxidant enzyme activity, and expression of salt response genes (Sharma et al., 2013). Additionally, *Bacillus pumilus* has been found to improve rice tolerance to the combined stresses of NaCl and high boron. This is due to its ability to limit the uptake of Na^+^ (Khan et al., 2016). Similarly to traditional exogenous substances, poly-γ-glutamic acid, as a microbe-secreted isopeptide, increased stress tolerance in *Brassica napus* seedlings by activating an H_2_O_2_ burst and subsequent crosstalk between H_2_O_2_ and Ca^2+^ signaling (Lei et al., 2017). Furthermore, low molecular weight secreted peptides from Brassicaceae have been found to stimulate the expression of salt stress-responsive elements (Yu et al., 2020). That demonstrates the potential of exogenous plant-derived peptides to help plants resist salt stress.

Maize (*Zay mays* L.) holds significant economic importance globally, yet its production is increasingly challenged by climatic changes and soil salinization (Hassani et al., 2021). In response to environmental stress, plants activate their defense mechanisms, resulting in the accumulation of secondary metabolites, including lipids, terpenoids, ketones, and alkaloids (Omoto et al., 2016; Jan et al., 2021). Metabolomic technology has revolutionized the study of low molecular weight compounds in organisms or cells, facilitating the identification of diverse metabolites and metabolic pathways involved in plant cell activities and secondary network metabolism (Liu et al., 2022; Wei et al., 2023). Metabolomics studies have revealed increased levels of metabolites such as amino acids and organic acids under salt stress compared to unaffected maize (Mo et al., 2023). In the previous study, metabolomic analysis of maize seedlings under salt stress identified three up-regulated peptides, nine down-regulated peptides and amino acids with Variable Importance in the Projection (VIP) values greater than 1 and *p* values less than 0.05 (**Supplementary Table S1**) (Wang, 2020). To evaluate their impact on plant height under salt stress, three up-regulated peptides were sprayed on maize seedlings at a concentration of 1×10^-7^ mol/L. The results revealed that maize seedlings treated with Valine-Threonine-Isoleucine-Aspartic acid (VTID) showed improved recovery from salt treatment and displayed enhanced growth compared to untreated maize seedlings.

The objective of this study was to assess the effects of VTID supplementation on salt-stressed maize seedlings. To achieve this, we examined the activity of antioxidant enzymes, levels of phytohormones, and osmoregulators. These factors play a crucial role in mitigating the harmful effects of reactive oxygen species (ROS) during salt stress. Furthermore, metabolomics and correlation analysis were conducted to identify key metabolites produced by VTID supplementation in salt-stressed maize seedlings. Taken together, our findings indicate that supplementation with VTID has the potential to promote the growth of salt-stressed maize seedlings. This effect is achieved by enhancing the activity of antioxidant enzymes and facilitating the production of isodolichantoside, a monoterpene indole alkaloid.

## 2 Materials and methods

### 2.1 Plant culture and treatment

VTID compound used in this study was synthesized by Bioengineering Co. Ltd (Shanghai, China). It has a relative molecular weight of 446.25 g/mol and a purity of 95%. To prepare a stock solution with a concentration of 1.12 mM, 0.5 mg of VTID was dissolved in 1 mL of double-distilled H_2_O (ddH_2_O). The maize seeds (Ningdan 33) used in this experiment were purchased from the local market in Ningxia, China.

Maize seeds were surface disinfected using 0.01% HgCl_2_ for 40 s, followed by rinsing thrice with sterile water (Kohli et al., 2019). The disinfected seeds were then sown on 0.8% agar medium containing Hoagland modified nutrient salts solution (NS10205-50mL, Beijing Coolaber Technology Co., Ltd, Beijing, China) and placed at 28°C with a relative humidity of 75% in an environmental chamber (Ji et al., 2022). Once the seedlings’ roots reached a length of 3 cm, they were transferred to plastic hydroponic bottles embedded in the sterile stones. For the control group, maize seedlings were irrigated with 1/2 Hoagland nutrient solution. In contrast, 1/2 Hoagland nutrient solution containing 100 mM NaCl was used for the salt stress group as described by Zhao et al. (2010).

**Figure 1:**
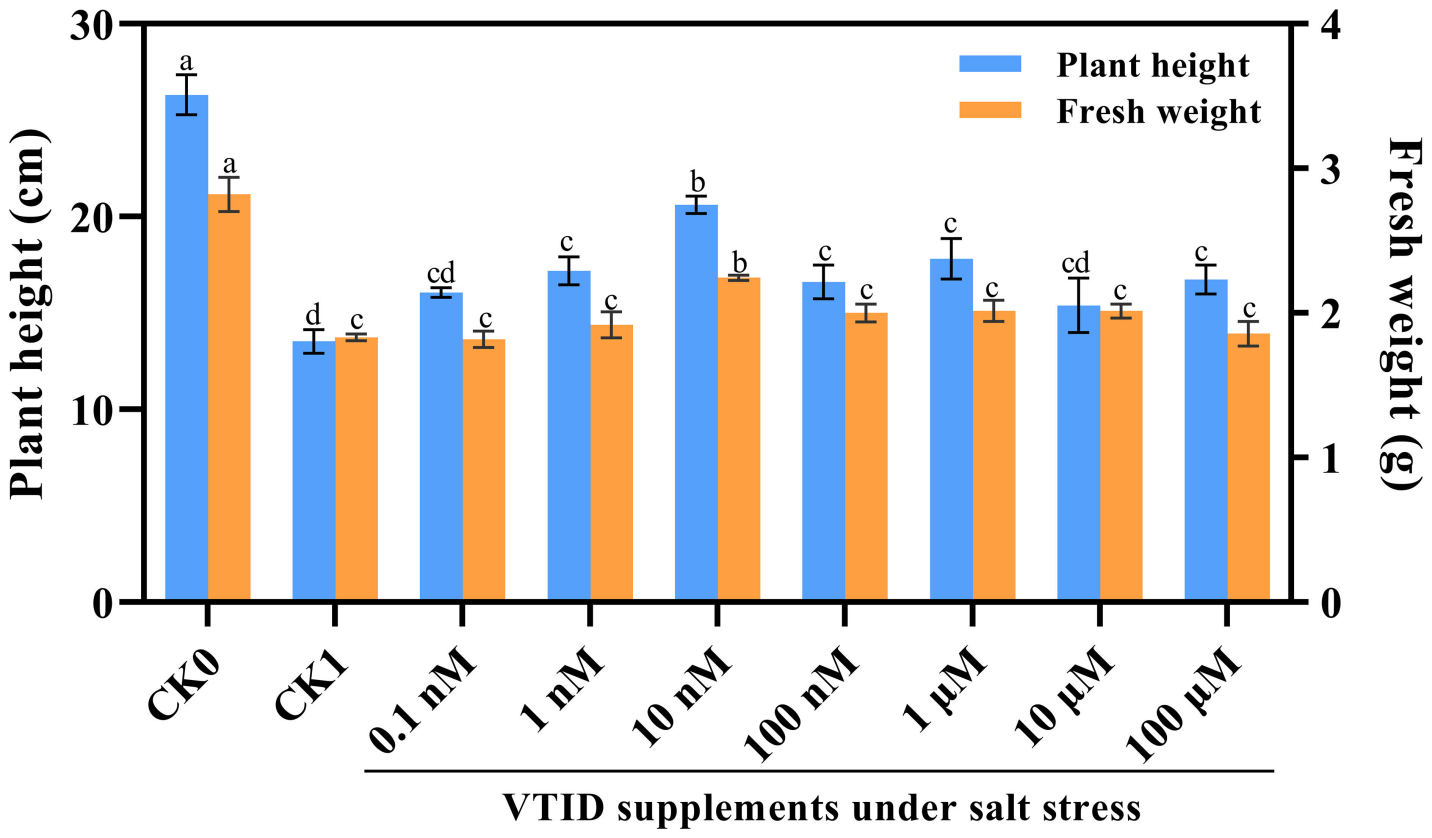
Plant height and fresh weight of maize seedlings under different treatments. CK0, maize seeds immersed in ddH_2_O and grew under non-salt stress conditions; CK1, maize seeds immersed in ddH_2_O and grew under salt stress conditions; 0.1 nM, 1 nM, 10 nM, 100 nM, 1 μM, 10 μM, and 100 μM indicated maize seeds immersed into different concentrations of VTID and grew under salt stress conditions.

Initially, various concentrations of VTID (i.e., 0.1 nM, 1 nM, 10 nM, 100 nM, 1 μM, 10 μM, and 100 μM) were supplemented with 1/2 Hoagland nutrient solution to determine the optimal concentration for plant growth under salt stress. It was observed that the addition of 10 nM VTID partially restored the growth of seedlings, particularly in terms of plant height and fresh weight (**Figure 1**). Subsequently, four treatments were implemented as follows: (i) CK0, ddH_2_O addition and non-salt stress treatment; (ii) T0, 10 nM VTID addition and non-salt stress treatment; (iii) CK1, ddH_2_O addition and salt stress treatment; (iv) T1, 10 nM VTID addition and salt stress treatment. After 12 days’ treatment, the aboveground part of seedlings were collected and stored at -80°C for subsequent analysis. The maize seedlings were cultivated at 28°C with a light-dark cycle of 16 hours of light and 8 hours of darkness, maintaining a relative humidity of 75%.

### 2.2 Determination of physiological and biochemical parameters in salt-stressed maize seedlings

After 12 days of treatment, the second leaf of the maize seedlings was collected to determine their physiological and biochemical indices. The activity of antioxidant enzymes, namely SOD, CAT, and POD, was determined using spectrophotometry according to the methodology outlined by Tian et al. (2024). In addition, the levels of MDA, soluble sugar, and proline were measured. The evaluation followed the instructions provided by Solarbio Science & Technology Co., Ltd. (Beijing, China), employing specific assay kits: SOD activity assay kit (BC0170), CAT activity assay kit (BC0200), POD activity assay kit (BC0090), MDA content assay kit (BC0020), plant soluble sugar content assay kit (BC0030), and proline content assay kit (BC0290).

To measure phytohormone levels, the collected leaves were pretreated to extract IAA, abscisic acid (ABA), and gibberellic acid (GA). Subsequently, enzyme-linked immunosorbent assay (ELISA) was performed using the IAA ELISA kit (MM-0953O2), ABA ELISA kit (MM-1185O2), and GA ELISA kit (MM-0125O2) following the instructions provided by Meimian Industrial Co., Ltd. (Jiangsu, China). This methodology was previously described by Ji et al. (2022).

### 2.3 Metabolite extraction

A 50 mg sample of the second leaf was collected and placed in a 2 mL Eppendorf tube containing 6 mm diameter grinding beads. Subsequently, 400 μL extraction buffer (methanol: water = 4:1, v/v) containing an internal standard (L-2-chlorophenylalanine at a concentration of 0.02 mg/mL) was added to the tube. The samples were ground using a Wonbio-96c frozen tissue grinder (Shanghai Wanbo Biotechnology Co., Ltd, Shanghai, China) for 6 min at -10°C and 50 Hz. Following grinding, low-temperature ultrasonic extraction was performed for 30 min at 5°C and 40 kHz. Afterward, the samples were stored at -20 °C for 30 min and subsequently centrifuged at 4°C at a speed of 13000 g for 15 min. The supernatant was then transferred into a new tube for further analysis.

### 2.4 Metabolite analysis by ultra-high performance liquid chromatography equipped with quadruple exactive mass spectrometer

Ultra-high performance liquid chromatography equipped with quadruple exactive mass spectrometer (UHPLC-QE-MS) was used for metabolomic profiling according to the methods described by Xiong et al. (2023). Separation was achieved using an ACQUITY HSS T3 column (100 mm×2.1 mm i.d., 1.8 μm; Waters, USA). The UPLC system was connected to a Thermo UHPLC-Q Exactive Mass Spectrometer with electrospray ionization (ESI) operating in both positive (3500 V) and negative (-2800 V) modes (He et al., 2023). As described by del Mar Gómez-Ramos et al. (2015), full scan data was obtained at a resolution of 70,000, and MS/MS data at a resolution of 17,500. Data acquisition utilized the Data Dependent Acquisition (DDA) mode over a mass range of 70-1050 m/z. The metabolomics data presented in the study are deposited in the MetaboLights repository, accession number MTBLS9653.

### 2.5 Data analysis

To assess the impact of VTID addition under salt stress and non-salt stress conditions, differences in maize height, fresh weight, SOD, CAT, and POD activities, MDA, IAA, GA, ABA, soluble sugar, and proline contents were analyzed. Analysis of variances (ANOVA) was employed to determine the significance (*p* < 0.05), followed by Tukey’s multiple comparison tests to assess the significance of the observed differences. Statistical analysis was performed using the IBM SPSS statistics (SPSS Inc., Chicago, IL, USA) and graphical visualization was performed using GraphPad Prism 8.0 (GraphPad Software, Boston, MA, USA).

The data obtained from UHPLC-QE-MS was processed using Progenesis QI software (Waters Corporation Milford, USA). The resulting metabolites were identified using HMDB (http://www.hmdb.ca/) and Majorbio database (Ren et al., 2022). Pairwise comparisons revealed differential metabolites (DMs) between the experimental groups. Statistical analysis including principal component analysis (PCA), and orthogonal partial least squares discriminant analysis (OPLS-DA) was performed by R package (Version 1.6.2). Significant DMs were determined based on a VIP score greater than 1 and an adjusted *p-*value below 0.05. To understand the biochemical pathways associated with DMs, metabolite pathway enrichment analysis (MPEA) was conducted using the Kyoto Encyclopedia of Genes and Genomes (KEGG) database (http://www.genome.jp/kegg/). ROC (Receiver Operating Characteristic) and linear regression analysis were performed through the free online platform of Majorbio cloud platform (cloud.majorbio.com).

## 3 Results

### 3.1 The optimal concentration of VTID used for maize seedlings under salt stress

To determine the optimal concentration of VTID supplementation, different concentrations of VTID were applied to salt-stressed maize seedlings. Initially, there was an increase in both plant height and fresh weight, followed by a subsequent decrease (**Figure 1**). Following extensive testing, it was discovered that the application of 10 nM VTID yielded the most remarkable results. This concentration led to a significant increase in plant height by 152.29% compared to salt-stressed seedlings (**Figure 1**). Furthermore, only the treatment with 10 nM VTID restored the fresh weight of maize seedlings to 2.24 g under salt stress. Conversely, the differences in plant height and fresh weight with and without VTID addition were not significant under non-salt stress. As a result, it can be concluded that the optimal concentration of 10 nM VTID significantly enhances the growth of maize seedlings under salt stress.

### 3.2 The effect of VTID on plant physiological attributes of maize seedlings under salt stress

**Figure 2:**
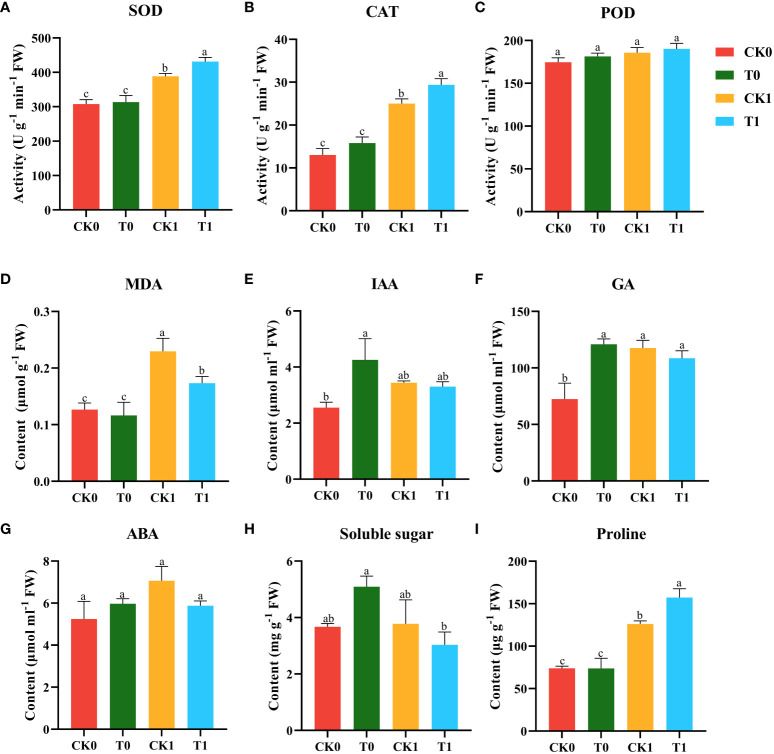
SOD **(A)**, CAT **(B)**, and POD **(C)** activities, MDA **(D)**, IAA **(E)**, GA **(F)**, ABA. **(G)**, Soluble sugar **(H)** and Proline **(I)** contents of maize seedlings under the different treatments (CK0, T0, CK1, and T1). CK0, ddH_2_O addition and non-salt stress treatment; CK1, ddH_2_O addition and salt stress treatment; T0, 10 nM VTID addition and non-salt stress treatment; T1, 10 nM VTID addition and salt stress treatment. Data are presented as the mean ± SEM. Bars labeled with different letters indicate a significant difference between treatments.

Environmental stresses led to the accumulation of ROS within plant cells, resulting in lipid peroxidation as indicated by MDA content. Under normal growth conditions without salt stress, the treatments with or without 10 nM VTID showed no significant changes in the activities of SOD, CAT, and POD, or the level of MDA (**Figures 2A–D**). Compared to salt stress without VTID, the exogenous application of VTID increased SOD and CAT activities by 10.9% and 17.3%, respectively, and significantly reduced MDA content by 32.7% (**Figures 2A, B, D**). There were no significant changes observed in POD activity among the four treatments (**Figure 2C**).

Phytohormones play a crucial role in plant growth adaptation under salt stress. To investigate the effect of VTID on maize seedlings under salt stress, the levels of IAA, GA, and ABA were tested. The exogenous VTID treatment resulted in an increase in the IAA content in maize seedlings under non-salt stress, but no significant difference was observed under salt stress (**Figure 2E**). The addition of VTID (T0 group), salt stress treatment (CK1 group), together with VTID addition and salt stress treatment (T1 group), showed an increase in the GA content in maize seedlings compared to those of blank treatment (CK0 group) (**Figure 2F**). However, VTID had no significant effect on ABA levels in maize seedlings under any growth conditions (**Figure 2G**).

Osmoregulatory substances such as soluble sugars and proline play a crucial role in reducing osmotic potential and enhancing water uptake or retention capacity in cells during salt stress (Choudhary et al., 2023). Under non-salt stressed conditions (CK0 and T0 groups), VTID had no significant effect on soluble sugars and proline contents of maize seedlings (**Figures 2H, I**). However, under salt stress conditions, proline level increased 1.25-fold in maize seedlings treated with VTID (T1 group) compared to those without VTID addition (CK1 group) (**Figure 2I**). VTID did not significantly impact soluble sugars under salt stress in maize seedlings. In conclusion, VTID supplementation increased the activity of antioxidant enzymes (SOD and CAT) while reducing the level of MDA in maize seedlings under salt stress.

### 3.3 Metabolic profiles of maize seedlings under salt stress and non-salt stress conditions with or without VTID treatment

**Figure 3:**
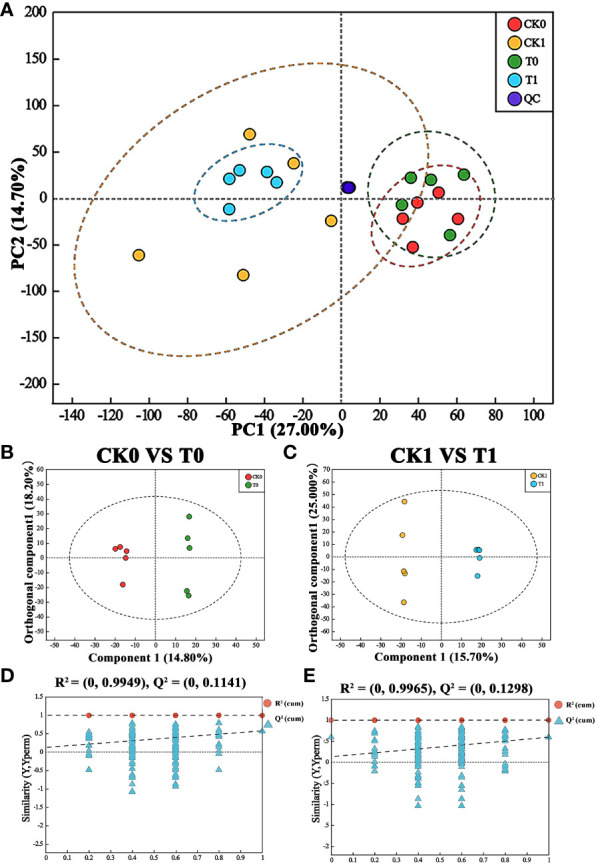
PCA model and OPLS-DA with corresponding values of R^2^X, R^2^Y, and Q^2^. **(A)** PCA score plot of CK0 (red circle), CK1 (yellow circle), T0 (green circle), T1 (blue circle), and QC samples (purple circle). **(B)** OPLS-DA score plot of CK0 (red circle) vs T0 (green circle). **(C)** OPLS-DA score plot of CK1 (yellow circle) vs T1 (blue circle). **(D, E)** Validation plot of the CK0 vs T0 (R^2^X = 0.489, R^2^Y = 0.992, and Q^2^ = 0.563) and CK1 vs T1 groups (R^2^X = 0.682, R^2^Y = 0.999, and Q^2^ = 0.593) obtained from 200 tests, respectively. CK0, ddH_2_O addition and non-salt stress treatment; CK1, ddH_2_O addition and salt stress treatment; T0, 10 nM VTID addition and non-salt stress treatment; T1, 10 nM VTID addition and salt stress treatment.

Metabolomic analysis was performed to gain insights into the responses of maize to salt stress with VTID supplementation. Principal component analysis (PCA) was conducted to visualize the distribution of metabolomics responses in the different treatments: CK0, T0, CK1, and T1. To ensure good reproducibility, any outliers with significant deviations were excluded from each treatment, and each treatment was performed with five biological replicates. PCA revealed distinct separation along the PC1 axis, accounting for 27.00% of the total variation between the salt-treated groups (CK1 and T1) and the non-stressed groups (CK0 and T0) (**Figure 3A**). Nevertheless, the PCA plot did not exhibit clear separations between the CK0 and T0 groups, as well as between the CK1 and T1 groups (**Figure 3A**). To enhance the detection of intergroup differences, we utilized the OPLS-DA statistical method, which has been proven to be more sensitive in cases where variables have low correlation (Xiao et al., 2022; Zheng et al., 2023). The OPLS-DA model revealed distinct separations between CK0 and T0 (R^2^X = 0.489, R^2^Y = 0.992, Q^2^ = 0.563, **Figures 3B, D**), as well as CK1 and T1 (R^2^X = 0.682, R^2^Y = 0.999, Q^2^ = 0.593, **Figures 3C, E**), ensuring the reliability of these results.

### 3.4 Classification of differential metabolites in VTID-treated maize seedlings under salt stress

**Figure 4:**
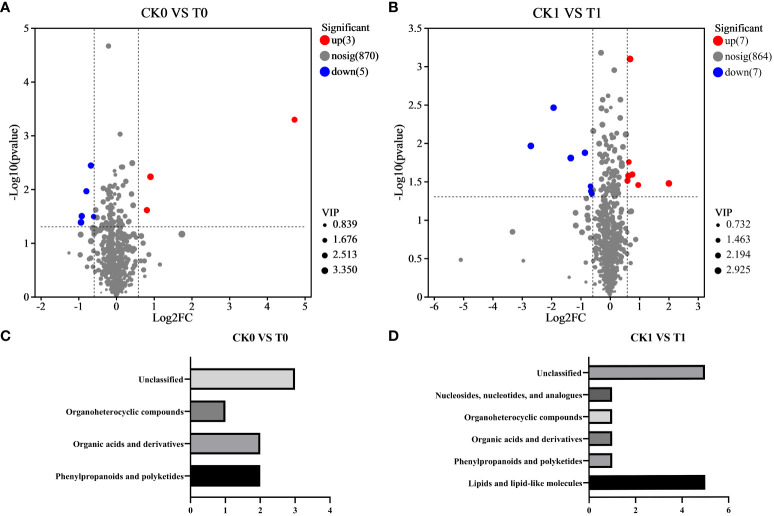
Volcano plot of significantly differential metabolites in CK0 vs T0 group **(A)** and CK1 vs T1 group **(B)**. Classification of differential metabolites in the CK0 vs T0 group **(C)** and CK1 vs T1 group **(D)** using HMDB categories, respectively. CK0 vs T0 group, ddH_2_O addition under non-salt stress and salt stress treatment; CK1 vs T1 group, 10 nM VTID addition under non-salt stress and salt stress treatment.

Differential metabolites were defined as significantly altered metabolites with a VIP threshold (VIP > 1) based on the OPLS-DA model. Generally, fold change (FC) > 1.5 and FC < 0.667 indicates the up- or down-regulated metabolites, respectively. In the CK0 vs T0 group, eight differential metabolites were screened, including three upregulated metabolites and five downregulated metabolites (**Figure 4A**; **Table 1**). These DMs are categorized into phenylpropanoids and polyketides, organic acids and derivatives, and organoheterocyclic compounds according to HMDB annotation (**Figure 4C**).

**Table 1:** Differential metabolites identified in the comparison between CK0 and T0 groups.

Number	Metabolite	HMDB Superclass	HMDB Class	Fold change
1	6,7-dihydroxy-3-(2-methylbut-3-en-2-yl)-2H-chromen-2-one	Phenylpropanoids and polyketides	Coumarins and derivatives	26.489
2	5-Hexyltetrahydro-2-oxo-3-furancarboxylic acid	Organoheterocyclic compounds	Lactones	1.757
3	Astin I	Organic acids and derivatives	Peptidomimetics	1.877
4	Euphodendroidin V	–	–	0.628
5	(1R)-Glutathionyl-(2R)-hydroxy-1,2-dihydronaphthalene	Organic acids and derivatives	Carboxylic acids and derivatives	0.578
6	5,7-dihydroxy-2-phenyl-2,3-dihydrochromen-4-one	–	–	0.660
7	Entandrophragmin	–	–	0.531
8	6-{[2-(3,4-dihydroxyphenyl)-4-oxo-6,8-bis[3,4,5-trihydroxy-6-(hydroxymethyl)oxan-2-yl]-7-[(3,4,5-trihydroxy-6-methyloxan-2-yl)oxy]-4H-chromen-5-yl]oxy}-3,4,5-trihydroxyoxane-2-carboxylic acid	Phenylpropanoids and polyketides	Flavonoids	0.523

-, Not applicable.

In the CK1 vs T1 group, 14 metabolites showed significant differences, with seven upregulated metabolites and seven downregulated (**Figure 4B**; **Table 2**). According to HMDB database analysis, nine differential metabolites in the CK1 vs T1 group were divided into five known categories. Among these, five metabolites were identified and annotated as lipids and lipid-like molecules (**Figure 4D**). Therefore, it can be concluded that lipids and lipid-like molecules are the most distinct metabolites according to the HMDB database analysis with the addition of VTID under salt stress.

**Table 2:** Differential metabolites identified in the comparison between CK1 and T1 groups.

Number	Metabolite	HMDB Superclass	HMDB Class	Fold change
1	Isodolichantoside	–	–	1.605
2	12S-HHTrE	–	–	1.562
3	PG(18:1(9Z)/0:0)	Lipids and lipid-like molecules	Glycerophospholipids	1.546
4	3-Deoxy-D-manno-octulosonate 8-phosphate	–	–	1.510
5	3,4,5-trihydroxy-6-({5-hydroxy-7-methoxy-4-oxo-2-phenyl-8-[3,4,5-trihydroxy-6-(hydroxymethyl)oxan-2-yl]-4H-chromen-6-yl}oxy)oxane-2-carboxylic acid	Phenylpropanoids and polyketides	Flavonoids	1.693
6	Daucic acid	Organic acids and derivatives	Hydroxy acids and derivatives	1.947
7	3-Hydroxytetradecanedioic acid	Lipids and lipid-like molecules	Fatty Acyls	4.016
8	Retrorsine	–	–	0.264
9	4-Hydroxyphenytoin glucuronide	Organoheterocyclic compounds	Azolidines	0.154
10	Succinyladenosine	Nucleosides, nucleotides, and analogues	Purine nucleosides	0.395
11	PS(22:5(4Z,7Z,10Z,13Z,16Z)/24:1(15Z))	Lipids and lipid-like molecules	Glycerophospholipids	0.553
12	Methyl (E)-2-dodecenoate	Lipids and lipid-like molecules	Fatty Acyls	0.631
13	PS(20:4(8Z,11Z,14Z,17Z)/24:0)	Lipids and lipid-like molecules	Glycerophospholipids	0.636
14	P-Coumaroylputrescine	–	–	0.651

-, Not applicable.

### 3.5 Screening of key differential metabolites in VTID-treated maize seedlings under salt stress

In the CK0 vs T0 group, 6,7-dihydroxy-3-(2-methylbut-3-en-2-yl)-2H-chromen-2-one was the most significantly upregulated metabolite, showing a remarkable FC of 26.489. Downregulated metabolites exhibited less variation in FC, as indicated in **Table 1**. The addition of VTID under salt stress conditions resulted in the change of more metabolites. Among the upregulated metabolites, 3-hydroxytetradecanedioic acid showed the highest FC, reaching 4.016 (**Table 2**). On the other hand, 4-hydroxyphenytoin glucuronide has the lowest FC among the downregulated metabolites, with a value of only 0.154.

**Figure 5:**
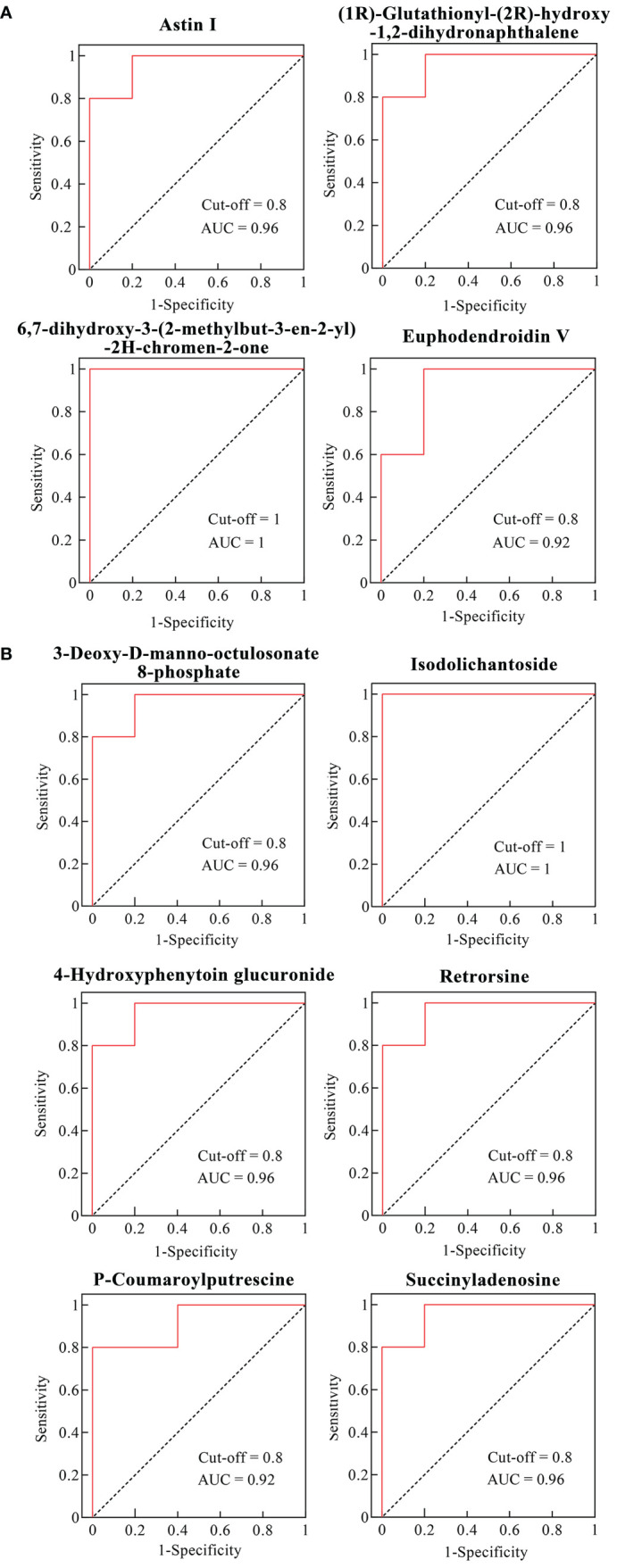
ROC curves of differential metabolites in the CK0 vs T0 group **(A)** and CK1 vs T1 group **(B)** with AUC > 0.9. CK0 vs T0 group, ddH_2_O addition under non-salt stress and salt stress treatment; CK1 vs T1 group, 10 nM VTID addition under non-salt stress and salt stress treatment.

To evaluate the predictive performance of the differential metabolites in VTID-treated maize seedlings, the ROC curve was employed for subsequent analysis. Previous studies showed that differential metabolites with an Area Under the Curve (AUC) value greater than 0.9 possess a higher potential to be utilized as biomarkers (Xia et al., 2013). In line with this, these results showed that four metabolites including astin I, 6,7-dihydroxy-3-(2-methylbut-3-en-2-yl)-2H-chromen-2-one, (1R)-glutathionyl-(2R)-hydroxy-1,2-dihydronaphthalene, and euphodendroidin V showed better performance in VTID-treated maize seedlings without salt stress (**Figure 5A**). Furthermore, under salt stress conditions, the AUC values of six out of 14 differential metabolites after VTID treatment were greater than 0.9, including 3-Deoxy-D-manno-octulosonate 8-phosphate, isodolichantoside, 4-hydroxyphenytoin glucuronide, retrorsine, P-coumaroylputrescine, and succinyladenosine. These findings suggest that these metabolites have the potential to serve as biomarkers for evaluating the impact of VTID on maize seedlings under salt stress conditions (**Figure 5B**) (Jiang et al., 2023).

### 3.6 Relationship between potential metabolite markers and plant growth parameters

To identify key metabolites that could predict sample differentiation after VTID treatment, ROC curve analysis was performed on the differential metabolites in the salt stress and non-salt stress groups, respectively. DMs with an AUC value greater than 0.9 were considered to have better predictive power. In the CK0 vs T0 group, four DMs with an AUC > 0.9 were identified, of which astin I and 6,7-dihydroxy-3-(2-methylbut-3-en-2-yl)-2H-chromen-2-one were upregulated by the addition of VTID. In the T1 vs CK1 group, six DMs with an AUC > 0.9 were found, including two upregulated metabolites, 3-deoxy-d-manno-octulosonate 8-phosphate and isodolichantoside.

**Figure 6:**
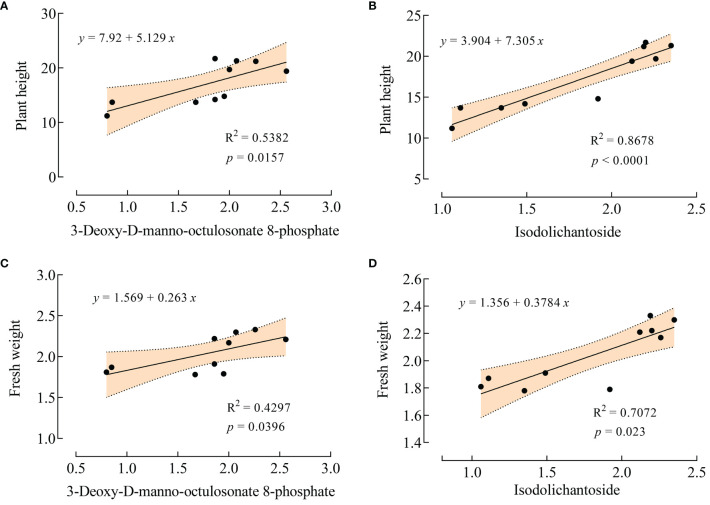
Linear regression analysis of 3-deoxy-D-manno-octulosonate 8-phosphate and isodolichantoside with plant height and fresh weight. **(A, B)** Linear regression of 3-deoxy-D-manno-octulosonate 8-phosphate **(A)** and isodolichantoside **(B)** with plant height, respectively. **(C, D)** Linear regression of 3-deoxy-D-manno-octulosonate 8-phosphate **(C)** and isodolichantoside **(D)** with fresh weight, respectively.

Plant height and fresh weight are commonly used to evaluate plant growth under salt stress. Interestingly, in this experiment, VTID treatment did not significantly affect the plant height and fresh weight of maize under non-salt stress conditions. This suggests that astin I and 6,7-dihydroxy-3-(2-methylbut-3-en-2-yl)-2H-chromen-2-one may have less correlation with these growth parameters in the T0 vs CK0 group. However, under salt stress, VTID resulted in a significant increase in plant height and fresh weight (**Figure 1**). Linear regression analysis revealed a strong relationship (R^2^ = 0.8678) between isodolichantoside and plant height, suggesting that isodolichantoside can account for the variation in plant height under salt stress conditions (**Figure 6B**). This suggests that VTID treatment may stimulate the accumulation of isodolichantoside, thereby promoting maize growth under salt stress conditions. However, the relationship between 3-deoxy-D-manno-octulosonate 8-phosphate and plant height was below 0.8, indicating that it could not effectively explain the variation in plant height under salt stress conditions (**Figure 6A**). For fresh weight, the goodness of fit between these two metabolites and fresh weight was below 0.8, indicating that they could not effectively predict the change in fresh weight (**Figures 6C, D**). In conclusion, the significant increase in maize plant height under salt stress caused by VTID treatment may be attributed to the accumulation of isodolichotoside.

## 4 Discussion

Salt stress is a major abiotic stress that adversely affects plant growth and development. It exerts a multifaceted impact on plants, disturbing critical processes including protein synthesis, enzyme activity, photosynthesis, leaf senescence, leaf growth, new leaf production, root growth, cell elongation, water uptake, seed germination, and overall yield reduction (Muchate et al., 2016; Xue et al., 2023). In response to this challenge, plants have evolved intricate biochemical mechanisms to mitigate the adverse effects. These biochemical mechanisms include activating antioxidant defense systems, developing osmotic tolerance, regulating hormonal levels, and more (Noreen and Ashraf, 2009; Yang and Guo, 2018). However, the excessive generation of ROS poses a significant threat, potentially leading to oxidative stress if the plant’s mechanisms for ROS detoxification are overwhelmed (Borges et al., 2023).

Previous studies have emphasized the effectiveness of exogenous substances including hormones, polyamines, nutrients, amino acids, sugars, and others, in aiding plants to alleviate damage caused by abiotic stress (Zhang et al., 2021). Among these, biological peptides, classified as amino acids, have emerged as pivotal players in alleviating abiotic stress in plants (Colla et al., 2015). Glutathione serves as a notable example in this context, as it functions to inhibit oxidative stress, stimulate enzymatic and non-enzymatic activities, and preserve cell membrane integrity (Punia et al., 2021). Similarly, systemin, a small peptide, has been found to enhance antioxidant capacity by upregulating enzymatic activity in plants subjected to salt stress (Cirillo et al., 2022). Another noteworthy compound is γ-aminobutyric acid (GABA), a non-protein amino acid that plays a crucial role in plant stress response. Under salt stress conditions, the expression of GABA-transaminase involved in GABA metabolism is significantly up-regulated in rice leaves (Kim et al., 2007). Similarly, when GABA was supplemented with salt-stressed maize, it partially restored seedling growth, even under moderate and severe stressed conditions (Wang et al., 2017). The combined application of GABA and potassium (K) to wheat has demonstrated notable benefits, including improved carbon and nitrogen assimilation, enhanced accumulation of osmotic regulators, reduced oxidative stress markers, and improved photosynthesis-related traits. These synergistic effects resulted in enhanced yield traits under salt stress compared to the application of either GABA or K alone (Kumari et al., 2023). In this study, VTID exhibited remarkable salt stress resistance by restoring the growth of maize seedlings even at a low concentration of 10 nM (**Figure 1**). Future investigations should explore whether VTID could yield even greater benefits when combined with other plant growth regulators, thereby enhancing stress tolerance and overall plant performance.

Salt stress induces the production of ROS, triggering membrane damage and lipid peroxidation, consequently elevating levels of MDA (Lodeyro et al., 2016). MDA concentration serves as a reliable indicator of lipid peroxidation in plants induced by environmental stress (Kong et al., 2016). To counteract the detrimental effects of ROS, plants have evolved an antioxidase system, which includes enzymes such as SOD, POD, and CAT. These enzymes play a vital role in scavenging ROS and reducing oxidative damage (Gill and Tuteja, 2010; Zhao et al., 2021). Proline, on the other hand, acts as an osmoregulator in plants, minimizing water loss and serving as a nitrogen source during plant recovery (Yi et al., 2022). It also enhances the activities of antioxidant enzymes (Ali et al., 2007; Devnarain et al., 2016). Our research findings demonstrate that under salt stress conditions, the application of exogenous VTID significantly enhances the activities of SOD and CAT, elevated proline levels, and reduced MDA accumulation. However, supplementation with VTID, with or without salt treatment, does not affect POD activity, possibly due to the concentration of NaCl used in maize (Pandey et al., 2015). These results suggest that VTID may function similarly to PGRs in alleviating salt stress-induced cell membrane damage, leading to improved salt tolerance in plants.

Phytohormones also play a crucial role in regulating plant development and enhancing tolerance to salt stress. Maintaining optimal concentrations of IAA, GA, and ABA can alleviate salt stress-induced oxidative damage to plant cells (Ryu and Cho, 2015). However, our research findings indicate that exogenous VTID does not significantly affect the accumulation of IAA, GA, and ABA in maize leaves under salt stress. This suggests that while phytohormones play a crucial role in other plant responses to stress, other metabolites in VTID might predominantly contribute to enhancing plant growth in the presence of salt stress. Metabolomic analysis of VTID-treated plants under salt stress conditions revealed 14 metabolites, of which five were unclassified and five were classified into lipids and lipid-like molecules. As an essential component of plant biological membranes, changes in membrane lipid composition in plants under salt stress can alter the activity of membrane proteins and the membrane permeability to water, ions, and metabolites (Guo et al., 2022). This explains the observed alterations in lipid and lipid-like molecules in maize seedlings in response to salinity upon supplementation with VTID.

Subsequent analysis involved a linear regression correlating plant growth indicators with predictive metabolites obtained from ROC curve analysis. Remarkably, a robust relationship (R^2 =^ 0.8678, *p* < 0.0001) was observed between isodolichantoside and plant height, indicating that isodolichantoside could elucidate the variation in plant height under salt stress conditions. Isodolichantoside, categorized as a monoterpene indole alkaloid (MIA), is a natural product isolated from *Psychotria correae*, *Cephaelis correae*, and *Strychnos mellodora* (Achenbach et al., 1993, 1995). Interestingly, isodolichantoside was previously unidentified in maize. Isodolichantoside isolated from leaf extract of *P. correae*, showed activity in the brine shrimp lethality test (Achenbach et al., 1995). In addition, MIAs are plant-natural products with important pharmacological, antioxidant, anti-inflammatory, anti-tumoral, immunomodulatory, antiviral, antimicrobial, and antiprotozoal activities (Matsuura et al., 2013). ROS, particularly H_2_O_2_, triggered by abiotic stress stimulates alkaloid biosynthesis and accumulation. This process serves as part of the plant’s antioxidant defense, helping restore an appropriate oxidative balance (Matsuura et al., 2014). From this, it can be concluded that VTID stimulates the accumulation of isodolichantoside to scavenge ROS and help plants survive under salt-stress conditions.

## 5 Conclusion

The present study investigated the effects of applying exogenous 10 nM VTID to maize seedlings under salt stress. The results revealed that the application of VTID increased proline levels, SOD and CAT activities, while decreasing MDA levels. These changes ultimately resulted in an increase in plant height and fresh weight. Metabolomics analysis indicated that VTID-induced DMs were associated with lipids and lipid-like molecules under salt stress conditions. Notably, a key metabolite, isodolichantoside showed a strong positive correlation with the height of maize seedlings, suggesting its involvement in the regulation of maize growth under salt stress conditions. Further research on short peptide VTID will provide valuable insights into its potential and will expand our current understanding of how plants regulate resistance to salt stress.

## Data availability statement

The data presented in the study are deposited in the MetaboLights repository, accession number MTBLS9653.

## Author contributions

KW: Investigation, Methodology, Writing – original draft. XL: Validation, Visualization, Writing – original draft. XZ: Conceptualization, Formal analysis, Funding acquisition, Methodology, Project administration, Supervision, Writing – review & editing. GY: Writing – review & editing. HW: Investigation, Writing – original draft. YX: Funding acquisition, Writing – review & editing. SI: Writing – review & editing. HJ: Methodology, Writing – review & editing. YQ: Methodology, Writing – review & editing. WG: Conceptualization, Formal analysis, Funding acquisition, Methodology, Project administration, Supervision, Writing – review & editing.
