# Supplementary material for: Metabolomics analysis reveals enhanced salt tolerance in maize through exogenous Valine-Threonine-Isoleucine-Aspartic acid application
Source: Front Plant Sci. 2024 May 17;15:1374142. doi: 10.3389/fpls.2024.1374142 (PMC11140139; doi:10.3389/fpls.2024.1374142)
Supplement: Supplementary file 1 [file Table_1.docx]

Table S1 Metabolomics analysis unveils differential short peptide and amino acid content in salt-stressed maize seedlings compared to non-salt-stressed seedlings

| Metabolite | VIP | Fold Change |
| --- | --- | --- |
| Ile-Ile-Glu-His | 2.4449 | 4.362 |
| Val-Thr-Ile-Asp | 2.2521 | 1.7828 |
| Arg-Gln-Arg-Gly | 3.0968 | 0.7706 |
| Asp-Lys-Pro-Leu | 2.2186 | 0.7582 |
| DL-Phenylalanine | 4.8029 | 0.6674 |
| L-Glutamate | 1.6658 | 0.5546 |
| L-Methionine | 1.572 | 0.5317 |
| Thr-Thr-Val-Lys | 1.4967 | 0.5114 |
| L-Tyrosine | 2.4688 | 0.5112 |
| L-Valine | 2.4943 | 0.4826 |
| L-Glutamine | 1.0579 | 0.4737 |
| Leu-Ala-Pro-Lys-Ile | 2.016 | 0.2051 |
